# Prevalence and Factors Associated With Suicidal Ideation in Medical Students With Migraine

**DOI:** 10.3389/fpsyt.2021.683342

**Published:** 2021-10-21

**Authors:** Jia-Ming Luo, En-Zhuo Liu, Hao-Di Yang, Cheng-Zhao Du, Li-Jie Xia, Zhi-Chao Zhang, Tong Li, Jia-Jun Ren, Jia-Qi Tang, Pei-Qi Tang, Yuan-Ru Tang, Shuang Zhu, Nisha Bhattarai, Anup Bhetuwal, Sheng-Xiong Pu

**Affiliations:** ^1^School of Psychiatry, North Sichuan Medical College, Nanchong, China; ^2^Department of Neurology, Affiliated Hospital of North Sichuan Medical College, Nanchong, China; ^3^School of International Education and Cooperation, North Sichuan Medical College, Nanchong, China

**Keywords:** migraine, suicidal ideation, medical college students, psychiatry comorbidities, associated factors

## Abstract

**Background:** The association between migraine and suicide ideation has been identified. However, the predictive factors of suicidal ideation are still controversial and whether migraine with aura can serve as an independent associated factor is uncertain. This manuscript studied the association between migraine with aura and suicidal ideation and explored the predictive factors for suicidal ideation.

**Methods:** We surveyed 9,057 medical students and included 579 medical students with migraine into our study population. All students completed the General Situation Questionnaire, the Verified Headache Questionnaire, Hamilton Anxiety Scale (24 items), Hamilton Depression Scale (24 items), 36-item Health Survey Brief (SF-36), Headache Impact Text-6 (HIT-6), Test Anxiety Scale (TAS), and Pittsburgh Sleep Quality Index (PSQI). Suicidal ideation was measured by the Self-rating Idea of Suicide Scale (SIOSS).

**Results:** Out of the 579 migraine medical college students, 562 (age 19.6 ± 1.6; 448 women and 114 men) were included in the final study. The positive rate of suicidal ideation was 13.7%. Compared with students suffering from migraine without aura, those having migraine with aura had higher suicidal ideation (*p* < 0.015). After adjusting for demographic factors and headache characteristics, migraine with aura was found to be independently associated with suicidal ideation. Other independent associated factors include anxiety, depression, test anxiety, sleep, headache, and quality of life. Among these various factors, high quality of life was found to play a protective role against suicidal ideation.

**Conclusions:** Migraine with aura is independently associated with suicidal ideation. Furthermore, anxiety, depression, text anxiety, poor sleep quality, and headache frequency are associated with suicidal ideation among medical college students with migraine.

## Introduction

Migraine is one of the most common types of primary headache with the prevalence of 10–18% (1) globally (2–4). Studies have shown that chronic pain may increase the frequency of thoughts of ending one's life (5). Psychogenic pain is significantly associated with increased suicidal ideation in all types of pain, especially migraine (6, 7). In addition, in the past few years, the burden of disease caused by migraine has made people more concerned about their consequences, such as the appearance of suicidal ideation (8).

Suicide has occurred more frequently in colleges and universities in recent years, making it a major public issue (9). For adolescence, suicide remains a leading cause of mortality worldwide (10). Suicidal behavior includes suicidal ideation, suicide plan, and suicide attempt. Among them, suicidal ideation is the most sensitive predictor (3). A meta-analysis of 34,904 college students showed that the overall prevalence of migraines among them was 16.1% (11), indicating that the college population is heavily affected by migraine. Migraine, which is the third most common disability factor, can undermine learning ability and life quality by severely affecting patients' social functions (12, 13). Once it affects the mental and psychological health of patients (14), the suicide risk rises, bringing greater social impacts. Therefore, it is necessary to explore the risk factors of suicidal ideation related to migraine.

Previous studies have shown the relationship between migraine and suicide (8, 15), but the suicidal ideation among each subtype of migraine remains controversial (5, 8). In 1992, Breslau et al. reported that migraine with aura had a higher rate of suicidal ideation (16). Wang et al. found a relationship between migraine with aura and suicidal ideation among adolescents aged 13–15 (5). However, they did not consider whether migraine with aura alone is independently associated with suicidal ideation after adjusting non-specific headache factors (headache frequency, headache intensity, and the duration of headache), and psychiatric comorbidities. Moreover, migraine is often accompanied by various psychiatric complications, which also affect the prognosis of this disease (17–19). Therefore, it is crucial to investigate the confounding factors, which will be done in this paper by exploring the relationship between migraine and suicidal ideation, as well as the related factors.

## Methods

### Study Design

This study was part of a cross-sectional survey carried out on the North Sichuan Medical College Students Headache Survey, aiming to determine the epidemiologic profile of headache and the relationship between psychiatric disorders and headache. This study consisted of multiple self-administered and other-rated scales with one-to-one information collection by a research group member from a medical college in Sichuan, China, from July 2018 to July 2019. This study was approved by the ethics committee of the Affiliated Hospital of North Sichuan Medical College and was filed on the clinical research platform of West China Hospital (No. NSMC20170420). All participants signed an informed consent form. This research is voluntary, confidential, practical, and non-harmful. Participants can choose to withdraw from the study at any time without any reason provided.

### Data Collection

The ID-Migraine was used to conduct a preliminary screening of migraine cases at a medical college in Sichuan Province, China. A total of 9,057 ID-Migraine screening questionnaires were issued, and 8,783 valid questionnaires were retrieved, with an effective response rate of 96.97%. We evaluated all valid questionnaires, of which 1,037 were initially screened positive for migraine.

The members of our research team were divided into many groups (Each group was trained with long-term professional knowledge and passed relevant tests, which ensured that each research group member can independently complete one-to-one information collection in accordance with unified standards). The group members asked students with positive initial screening result for headaches *via* telephone or email if whether they are willing to give basic information about themselves and headaches. During the collection, the information was recorded in strict accordance with uniform criteria, and all information was kept confidential.

A total of 1,007 students participated in further research information collection. Should there be any information missing, we made another telephone call or wrote an email to complete the collection. Next, these students were diagnosed by two or more specialized neurologists according to the diagnostic criteria and the classification of ICHD-3 (14).

**Figure 1:**
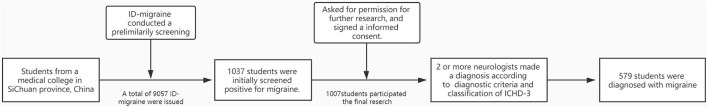
The screening process of medical students with migraine.

Finally, 579 students were diagnosed with migraines (The flow chart diagram is shown in Figure 1).

### Target Group

In China, over 90% of medical students attend public universities and the rest attend private universities. After screening for ID-Migraine, we had 579 students suffering from migraine out of 8,783 participants. The overall prevalence of migraine was slightly lower than medical students in Northern China (8.6%) and Suzhou, China (7.91%). Moreover, it was lower than the global prevalence of migraine (10–18%). Thus, our study cohort is generally representative of the sample obtained from the medical students.

### Headache Diagnosis

The ID-Migraine was used for the screening of headaches. Migraine can be further divided into migraine with aura (MA) and migraine without aura (MO), and the diagnosis is based on the ICHD-3 version (20). Exclusion criteria are as follows: students with secondary headaches, other chronic diseases, anxiety, depression, or other mental illnesses clinically diagnosed before the onset of headache; students who were taking medications for migraine, anxiety, or depression; and students who refused to sign the consent form.

### Questionnaires

1. **Demographic data:** name, sex, nation, age, height (cm), weight (kg), grade, major, living expenses(¥)/month, smoking history (smoke>1/day, time>1 year), drinking history (drinking in the past year), genetic history of migraine.
2. **A valid screening questionnaire for headaches:** the final three-item screening questions of the ID-Migraine (Pfizer Inc., New York, NY, USA) test are as follows: (1). During the last 3 months, did you feel nauseated or sick in your stomach when headaches strike? (2). Did light bother you when you had a headache (a lot more than when you do not have headaches)? (3). Did your headache restrict your ability to work, study, or do other things? The ID-Migraine is a very simple and valid tool, often used for the diagnosis of migraine (21–23). The sensitivity of ID-Migraine is 0.84, 95% CI 0.75–0.90, and the specificity of ID-Migraine is 0.76, 95% CI 0.69–0.83 (24). The overall sensitivity and specificity of the Chinese version of ID-migraine were 84 and 64% (25), respectively. If two or more of the answers are “yes,” the patient is possibly suffering from migraine and will be judged to have a positive screening result.
3. **A valid headache questionnaire:** based on the 3rd edition of *the International Classification of Headache Disorders ICHD-3*, the content of which includes the age of onset (years), the course of disease (years, the first diagnosis of headache-related diseases until this investigation), triggers (≥2 migraine attacks directly caused by this factor), the location of the headache, the nature of the headache, the duration of severe headache (hours, all time periods are divided into two categories:> 1 and <1 h), pain degree (VAS score, 0–10), the number of migraine days per month (or days, if the number of days is <1 day, it is counted as 1 day), aura symptoms 1 h before the headache, symptoms associated with the headache, and the use of painkillers.
4. **A valid self-rating idea of suicide scale:** we used the Self-rating Idea of Suicide Scale (SIOSS) to test. The scale has good reliability and validity for screening Chinese college students' suicidal ideation, with focus reliability of 0.86, an internal consistency coefficient of 0.79, and split-to-half reliability of 0.824 (26). It includes four factors: despair, suicide, optimism, and cover-up. Only “yes” or “no” are given as answers. If the cover factor is ≥ 4, the test is invalid. Generally, ≥12 score is taken as the critical value. The higher the score, the stronger the suicidal ideation.
5. **HAMA/HAMD (Hamilton Anxiety Scale/Hamilton Depression Scale):** the Hamilton Anxiety Scale and Hamilton Depression Scale have been widely used to assess the appearance and the severities of anxiety and depression (27, 28). In China, this scale is commonly used to screen anxiety and depression. In this study, all members of our research team received professional training from two psychiatrists, ensuring the consistency and professionalism of the inquiry and evaluation. In our preliminary evaluation from the Hamilton Depression Scale score, anxiety was determined if the score was > 7 points; depression if it is >8 points. In both scales, the higher the score, the severer the disorder.
6. **The PSQI (Pittsburgh Sleep Quality Index):** This scale test factors including subjective sleep quality, time to fall asleep, sleep efficiency, sleep disorders, sleeping medications, and daytime function. The higher the score, the poorer the sleep quality (29). In China, using a score of more than seven points to judge whether there is a sleep quality problem has good sensitivity and specificity (sensitivity and specificity are 98.3 and 90.3%, respectively) (30).
7. **The TAS (Test Anxiety Scale):** the new version of 37 items originally revised and compiled by Sarason on the basis of TAQ has effectively improved the sensitivity and reliability of the scale (31). The Chinese version was translated and used by Wang Caikang in 1999 (32). The scale includes tests on psychological feelings and physical discomfort before and after the test. A score of <12 is considered as low anxiety, 12–20 is considered as moderate anxiety, and 20 or more is considered as high anxiety.
8. **The HIT-6 (Headache Impact Text-6):** this scale evaluates the impact of headaches on the physical condition. Scores ≤49 represent little or no impact; scores between 50 and 55 represent impact to a limited extent; scores between 56 and 59 represent substantial impact; and scores ≥60 indicate severe impact (33).
9. **The SF-36 (36-item Health Survey Brief):** this scale contains eight health dimensions, which can also be categorized into physical health and mental health (34). The higher the 21 scores, the higher the quality of life.

### Statistical Analysis

Continuous variables that conform to normality and variance are expressed as x ± s (mean ± standard deviation), while those that do not conform are expressed as M (Q1, Q3)[mean (lower quartile, upper quartile)], and the categorical variables are expressed as the rate.

The *X*^2^-test (chi-square test) was used to test the difference between the groups of the categorical variables. The Student *t* was used to compare the difference between the continuous variable groups that conform to normality and the uniform variance, while the Mann-Whitney *U*-test was used to compare the continuous variable groups (including: the scores of HAMA and HAMD, head frequency) that do not conform to the normality or uniform variance.

To assess the association of variables in Table 1 with suicidal ideation, univariable logistics regression analyses were performed with suicidal ideation as the dependent variable. All factors in Table 1 were entered as independent variables into the multivariable model to identify potentially predictive factors for suicidal ideation. The predictors for suicidal ideation were presented as odds ratios (OR) with 95% confidence intervals (95% CI).

**Table 1:** Characteristics of the study population.

**Variable**	**Migraine without aura**	**Migraine with aura**	***P*-value**
**Demographic characters**			
Age, mean (SD), years	19.6 ± 1.6	19.6 ± 1.5	0.701^⋆^
Sex, [*n* (%)], female	389 (79.9)	59 (78.7)	0.808^*^
BMI, *n* (%)			0.654^*^
Underweight	108 (22.2)	19 (25.3)	
Normal	332 (68.2)	46 (61.3)	
Overweight	32 (6.6)	7 (9.3)	
Obese	15 (3.1)	3 (4.0)	
Grade, *n* (%)			0.833^*^
Freshman	263 (54.0)	41 (54.7)	
Sophomore	135 (27.7)	23 (30.7)	
Junior	39 (8.0)	5 (5.3)	
Senior	50 (10.3)	7 (9.3)	
Consumption of tobacco *n* (%)	16 (3.3)	1 (1.3)	0.358 ^*^
Consumption of alcohol *n* (%)	23 (4.7)	3 (4.0)	0.781 ^*^
**Headache-related factors**			
Headache frequency, day/month, (QL, QU)	2 (0, 3.4)	2 (1, 4)	0.967**^Δ^**
Intensity, *n* (%)			0.475^*^
Low-degree	203 (41.7)	29 (38.7)	
Medium-degree	256 (52.6)	39 (52.0)	
High-degree	28 (5.7)	7 (9.3)	
Duration of severe headache, hours, n (%)			<0.001^*^
<1 h	246 (50.5)	57 (70.7)	
>1 h	241 (49.5)	22 (29.3)	
Positive family history of migraine, *n* (%)	188 (56.8)	30 (60.0)	0.670^*^
**Psychological factors**			
HAMA (QL, QU)	8 (4.96, 12)	10 (6, 16)	0.005**^Δ^**
HAMD (QL, QU)	11 (7, 11)	14 (9, 20)	0.002**^Δ^**
TAS, mean (SD)	18.4 ± 7.1	19.2 ± 6.6	0.393^⋆^
HIT-6, mean (SD)	54.1 ± 8.7	54.5 ± 9.4	0.724^⋆^
Suicidal ideation, n (%)			0.015^*^
Yes	60 (12.3)	17 (22.7)	
No	427 (87.7)	58 (77.3)	

*BMI, body mass index (Chinese standard value: underweight = BMI <18.5; normal = 18.5 ≤ BMI <24; overweight = 24 ≤ BMI <28; obese = BMI > 28)*.

*HAMA, Hamilton Anxiety Scale; HAMD, Hamilton Depression Scale; TAS, Test Anxiety Scale; PSQI, Pittsburgh Sleep Quality Index; HIT-6, Headache Impact Text-6; SF-36, 36-item Health Survey Brief*.

*n, numbers; SD, standard deviation; QL, lower quartile; QU, upper quartile*.

* *Chi-square test*.

⋆ *Independent-sample t- tests*.

Δ *Mann-Whitney U-test*.

In multivariable logistics regression, variables were divided into three models: (1) Model 1: demographic and social factors (Demo), including age, sex, grade, BMI, history of tobacco, or alcohol consumption (2); Model 2: headache-related factors (HF), including headache frequency, intensity, duration of migraine, family history of migraine; and (3) Model 3: psychological factors (PF), including HAMA, HAMD, TAS, HIT-6, PQSI, and SF-36 score. The multicollinearity among the predictive factors was checked using the variance inflation factor in which a value >10 was considered to have serious problem of collinearity.

The significance level was set to 0.05. Data were analyzed using the Statistical Package for the Social Sciences (Version 25.0 for Windows).

## Results

A total of 579 medical college students were diagnosed with migraine, including 114 male and 448 female (female/male= 3.9) with a mean age of 19.6 ± 1.6 years. Among them, 17 students were excluded because the questionnaires filled by them were incomplete. Therefore, 562 students with migraines were finally included in our research sample.

### Clinical Characteristics in Students With Migraine

Our study showed that the overall prevalence of suicidal ideation among students suffering from migraines was 13.7%. Table 1 presents the demographic information about the participants and illustrates the differences among the different migraine subtypes. The prevalence of suicidal ideation was much higher among students with migraine with aura than those without aura (*p* = 0.015). The proportions of those with suicidal ideation in migraine with aura and migraine without aura were 22.1 and 12.3%, respectively. Also, the table shows that, compared to migraine without aura, migraine with aura had a longer severe headache duration (*p* < 0.001) and higher score for HAMA (*p* = 0.005) and HAMD (*p* = 0.002).

### Factors Associated With Suicidal Ideation in Medical College Students

In Table 2, the results were obtained by using univariable logistic regression analysis to identify the factors associated with suicidal ideation. Among headache-related factors, only high-frequency headaches (OR = 1.2, 95% CI = 1.1–1.2), *p* < 0.01) associated with the suicidal ideation. Almost all the psychological factors had a significant association with suicidal ideation: high scores of HAMA (OR = 1.3, 95% CI = 1.2–1.3, *p* < 0.01), HAMD (OR = 1.2, 95% CI =1.13–1.21, *p* < 0.01), TAS (OR = 1.2, 95% CI = 1.2–1.3, *p* < 0.01), HIT-6 (OR = 1.1, 95% CI = 1.06–1.12, *p* < 0.01), and PSQI (OR = 1.4, 95% CI = 1.3–1.5, *p* < 0.01), as well as low SF-36 (OR = 0.9, 95% CI 0.87–0.92, *p* < 0.01) scores.

**Table 2:** Factors associated with suicidal ideation in students with migraine using univariable logistic regression analyses.

**Variable**	**OR (95% CI)**	***P*-value**
**Demographic characters**		
Age, years	0.9 (0.8–1.1)	0.313
Sex, female	1.829	0.09
**BMI**		
Underweight	1.4 (0.8–2.5) 0.211
Normal	Reference	
Overweight	1.6 (0.7–3.8)	0.306
Obese	1.4 (0.4–5.2)	0.573
**Grade**		
Freshman	Reference	
Sophomore	2.0 (1.2–3.4)	0.008
Junior	0.6 (0.2–2.0)	0.378
Senior	0.9 (0.4–2.3)	0.829
Consumption of tobacco	1.4 (0.4–4.9)	0.632
Consumption of alcohol	0.8 (0.2–2.8)	0.743
**headache-related factors**		
Headache frequency, day/month	1.1 (1.1–1.2)	<0.001
**Intensity**		
Low-degree	Reference	
Medium-degree	0.9 (0.6–1.5)	0.76
High-degree	1.6 (0.6–3.9)	0.336
Duration of severe headache, hours		0.629
<1 h	Reference	
>1 h	1.1 (0.7–1.8)	
Positive family history of migraine	1.2 (0.7–2.0)	0.471
Aura		0.017
No	Reference	
Yes	2.1 (1.1–3.8)	
**Psychological factors**		
HAMA	1.2 (1.2–1.3)	<0.001
HAMD	1.2 (1.1–1.2)	<0.001
TAS	1.2 (1.2–1.3)	<0.001
HIT-6	1.1 (1.06–1.12)	<0.001
PSQI	1.4 (1.3–1.5)	<0.001
SF-36	0.9 (0.87–0.92)	<0.001

*BMI, body mass index (Chinese standard value: underweight = BMI <18.5; normal = 18.5 ≤ BMI <24; overweight = 24 ≤ BMI <28; obese = BMI > 28)*.

*HAMA, Hamilton Anxiety Scale; HAMD, Hamilton Depression Scale; TAS, Test Anxiety Scale; PSQI, Pittsburgh Sleep Quality Index; HIT-6, Headache Impact Text-6; SF-36, 36-item Health Survey Brief*.

*CI, confidence interval; OR, odds ratio*.

### The Relationship Between Suicidal Ideation and Migraine With Aura, and the Predictive Factors for Suicidal Ideation

Table 3 shows the results of multivariable logistic regression analyses that identified the potential predictive factors of suicidal ideation in students with migraine. As shown in the table, when aura was added to the multivariate regression model as an explanatory variable for suicidal ideation (Table 3, unadjusted model), aura was associated with suicidal ideation. After adjusting for Demo, HF, and PF, the presence of an aura was independently predictive of suicidal ideation (OR = 2.8, 95% CI = 1.2–6.3, *p* = 0.014, Table 3, adjusted multivariable models 1, 2, and 3, respectively). The results showed that the presence of aura significantly predicted suicidal ideation. Other predictive factors are listed in Table 4. Except for migraines with aura, all variables were related with psychological factors. There were no clinical symptoms associated with headache that were independently predictive of suicidal ideation.

**Table 3:** Multivariable logistic regression analysis, demonstrating the independent association of aura on suicidal ideation.

	**Suicidal ideation**	
**Controlled covariates**	OR (95% CI)	*P*-value
Unadjusted	2.1 (1.1–3.8)	0.017
**Model 1:** adjusted for demo	2.0 (1.1–3.8)	0.024
**Model 2:** adjusted for demo and HF	2.2 (1.2–4.1)	0.015
**Model 3:** adjusted for demo and HF and PF	2.8 (1.2–6.3)	0.014

*CI, confidence interval; OR, odds ratio; Demo, demographics; HF, headache-related factors; PF, psychological factors. (Demo) includes: age, gender, grade, smoking, and drinking; headache-related factors (HF) include headache frequency, intensity, duration of migraine, and family history of migraine; psychological factors (PF) include the score of HAMA, HAMD, TAS, HIT-6, PSQI, and SF-36*.

**Table 4:** Multivariable logistic regression analysis of factors associated with suicidal ideation in students with migraine.

	**Suicidal ideation**			
	**OR (95% CI)**	**β**	**SE (β)**	***P*-value**
Aura	2.8 (1.2–6.3)	1.0	0.4	0.014
HAMA	1.1 (1.0–1.1)	0.1	0.03	0.048
HAMD	1.1 (1.0–1.1)	0.1	0.03	0.058
TAS	1.2 (1.1–1.3)	0.2	0.03	<0.001
HIT-6	1.1 (1.0–1.1)	0.1	0.02	0.012
PSQI	1.2 (1.1–1.4)	0.2	0.06	0.002
SF-36	0.95 (0.92–0.98)	−0.1	0.02	0.002

*CI, confidence interval; OR, odds ratio; HAMA, Hamilton Anxiety Scale; HAMD, Hamilton Depression Scale; TAS, Test Anxiety Scale; PSQI, Pittsburgh Sleep Quality Index; HIT-6, Headache Impact Text-6; SF-36, 36-item Health Survey Brief*.

## Discussion

It is widely acknowledged that migraine is associated with suicidal ideation. Besides, students with migraine have a higher prevalence of psychiatric comorbidities. However, the result obtained from our study is quite different from previous ones. The present study shows that migraine with aura is associated with suicidal ideation, after adjusting demographic information, head-related factors, and a variety of psychiatry comorbidities. In addition, the population of the present study is based on a large-scale of students and is strictly screened, which helps to reflect the adolescence's suicide problems better.

The incidence of suicidal ideation among the general population is between 20 and 30% (35), while that among patients with migraine ranges from 16.8 to 26.6% in Asia (5, 36, 37). The large range for the latter population may be due to the differences in suicidal ideation determination methods and questionnaires (questions, suicidal ideation questionnaire, and a question in depression scale). In addition, the lower percentage could be because our research subjects are college students knowing a lot of knowledge about life activities and possessing higher respect for life. Moreover, due to the mental and psychological theories they learn endow them with stronger self-efficacy to resist negative emotions, our research results are lower than those of the general migraine population (38). Studies have pointed out that people suffering from migraine with aura are more likely to have suicidal ideation than those with other types of migraine (5, 7, 15); our study also supports this viewpoint. Breslau et al. had shown that migraine with aura had a higher rate of suicidal ideation, but did not consider other confoundings (16). After the demographic factors and psychiatric comorbidities are combined, migraine with aura is still independently associated with suicidal ideation. This means that some factors or characteristics of migraines with aura may influence the prevalence of suicidal ideation. In China, medical college students usually undergo an arduous training and long-term curriculum which means they experience a higher mental and physical pressure. A study shows that compared with other subject areas, the study of medicine is associated with higher stress (39). Besides, a meta-analysis based on Chinese medical students refers that Chinese medical students have relatively high prevalence of depression, anxiety, and suicidal ideation (40). Thus, paying close attention to the psychiatry comorbidities, which could affect the prevalence of suicidal ideation, is essential. According to a study based on neural electrophysiology, migraine with aura has a higher cortical response amplitude and a lower threshold compared with other types of migraine (41). The level of 5-HT is related to migraine with aura and suicidal ideation (42), and aura itself is also related to the activation of the trigeminovascular system caused by cortical spreading depression (43, 44). At the same time, regarding the effect of factors associated with headache on suicidal ideation, some studies reported that pain is a risk factor for suicidal ideation, and chronic pain can increase the risk multiples (6). The Korean article infers that any type of headache accompanying strong headache or depression intensity might predispose the patient to suicidal ideation (45). It may be our study population based on the migraine population and does not include the healthy population; our results did not support this conclusion. Using univariable logistic regression analyses, it can be concluded that the frequency of headaches is associated with suicidal ideation, but after combining other variables, the conclusion does not stand. In short, neither demographic information nor headache-related factors independently associated with suicidal ideation in our study.

Migraine is often accompanied by psychiatric comorbidities (46), seriously affecting the prognosis of migraine and the occurrence of suicidal ideation. Our study found that migraine with aura is more associated with psychiatric comorbidities, which is similar to previous studies (7, 47). We also found that suicidal ideation of migraine with aura is higher than that without aura, and it independently associated with suicidal ideation. On this basis, we also conclude that students with migraine with aura experience more severe anxiety and depression, which may mean that the migraine with aura had a greater impact on their lives. Migraine, depression, anxiety, and suicidal ideation are all associated with high levels of 5-HT (2A) receptor in the prefrontal cortex and hippocampus of the nervous system (48), which may be the reason of their accompanying syndromes. According to a meta-analysis of polysomnography, sleep depth and continuity are associated with mental disorders, including anxiety (49). Based on previous studies, it can be speculated that the hormonal dysregulation and headache-related factors in migraine patients bring anxiety and depression, which in turn can affect the quality of sleep and lead to a decrease in the quality of life. At the same time, the chronic pain and the reduced pleasant experience are risk factors for suicidal ideation (50). College students face great academic pressure, especially a variety of exams, which can play the role of external environmental stimuli for migraine-related suicidal ideation (15). All kinds of factors adding together may eventually lead to the occurrence of suicide ideation among migraine college students.

Based on our study, we should pay more attention to migraine with aura and its psychiatry comorbidities, which may result in suicidal ideation. Antidepressants are the choice of many students for concomitant psychiatric comorbidities, and although they may slightly increase suicidal ideation (51), the overall advantages overweight limitations. In terms of drug selection, SSRIs can be used instead of venlafaxine, as there has been literature indicating that it can slightly increases suicidal ideation (52).

## Conclusion

Our results indicate that the prevalence of suicidal ideation among medical college students with migraine was 13.7%. Besides, migraine with aura is independently associated with suicidal ideation after adjusting headache-related factors and psychological factors. Many psychiatric comorbidities (anxiety, depression, low sleep quality, test anxiety, the severity of headache, and life quality) are the predictive factors of suicidal ideation. Moreover, the prevalence of suicidal ideation and duration of severe headache in students with migraine with aura is higher than those with migraine without aura.

## Data Availability Statement

The raw data supporting the conclusions of this article will be made available by the authors, without undue reservation.

## Ethics Statement

This study was approved by the Ethics Committee of the Affiliated Hospital of North Sichuan Medical College and was filed on the clinical research platform of West China Hospital, with the record number NSMC20170420. The patients/participants provided their written informed consent to participate in this study.

## Author Contributions

E-ZL was involved in the study design, data analysis, and the composing of this manuscript. J-ML provided the subject of this study and critically revised this manuscript. H-DY wrote the data collection part of this manuscript. C-ZD, L-JX, Z-CZ, and TL searched and reviewed the references. J-JR, J-QT, P-QT, Y-RT, and SZ collected the data. NB and AB modified this manuscript. All authors contributed to this manuscript and approved the submitted version.

## Funding

This work was financially supported by the Funding Project of the Bureau of Science and Technology and Intellectual Property of Nanchong City (No. NSMC20170420).

## Conflict of Interest

The authors declare that the research was conducted in the absence of any commercial or financial relationships that could be construed as a potential conflict of interest.

## Publisher's Note

All claims expressed in this article are solely those of the authors and do not necessarily represent those of their affiliated organizations, or those of the publisher, the editors and the reviewers. Any product that may be evaluated in this article, or claim that may be made by its manufacturer, is not guaranteed or endorsed by the publisher.
